# Suramin Inhibits Osteoarthritic Cartilage Degradation by Increasing Extracellular Levels of Chondroprotective Tissue Inhibitor of Metalloproteinases 3

**DOI:** 10.1124/mol.117.109397

**Published:** 2017-10

**Authors:** Anastasios Chanalaris, Christine Doherty, Brian D. Marsden, Gabriel Bambridge, Stephen P. Wren, Hideaki Nagase, Linda Troeberg

**Affiliations:** Arthritis Research UK Centre for Osteoarthritis Pathogenesis, Kennedy Institute of Rheumatology, (A.C., C.D., G.B., H.N., L.T.), Structural Genomics Consortium (B.D.M.), and Alzheimer’s Research UK Oxford Drug Discovery Institute (S.P.W.), University of Oxford, Oxford, United Kingdom

## Abstract

Osteoarthritis is a common degenerative joint disease for which no disease-modifying drugs are currently available. Attempts to treat the disease with small molecule inhibitors of the metalloproteinases that degrade the cartilage matrix have been hampered by a lack of specificity. We aimed to inhibit cartilage degradation by augmenting levels of the endogenous metalloproteinase inhibitor, tissue inhibitor of metalloproteinases (TIMP)-3, through blocking its interaction with the endocytic scavenger receptor, low-density lipoprotein receptor–related protein 1 (LRP1). We discovered that suramin (C_51_H_40_N_6_O_23_S_6_) bound to TIMP-3 with a *K*_D_ value of 1.9 ± 0.2 nM and inhibited its endocytosis via LRP1, thus increasing extracellular levels of TIMP-3 and inhibiting cartilage degradation by the TIMP-3 target enzyme, adamalysin-like metalloproteinase with thrombospondin motifs 5. NF279 (8,8′-[carbonyl*bis*(imino-4,1-phenylenecarbonylimino-4,1-phenylenecarbonylimino)]*bis*-1,3,5-naphthalenetrisulfonic acid hexasodium salt), a structural analog of suramin, has an increased affinity for TIMP-3 and increased ability to inhibit TIMP-3 endocytosis and protect cartilage. Suramin is thus a promising scaffold for the development of novel therapeutics to increase TIMP-3 levels and inhibit cartilage degradation in osteoarthritis.

## Introduction

Osteoarthritis (OA) is a common degenerative joint disease, in which cartilage degradation and subchondral bone remodeling cause pain and impaired movement of affected joints. The most common risk factors for OA are age, joint injury, and obesity, which all alter the mechanical environment of the joint and initiate catabolic joint remodeling. The disease is estimated to affect 10% of men and 18% of women older than 60 years of age (Woolf and Pfleger, 2003), and its incidence is predicted to rise with increasing population age and obesity. No disease-modifying drugs are currently available, and treatment is currently limited to management of symptoms by analgesia or joint replacement surgery. There is thus a significant clinical need for the development of novel therapeutic strategies.

Articular cartilage covers and protects the ends of bones in articulating joints, enabling smooth, frictionless joint articulation. Degradation of this cartilage layer is a key feature in the pathogenesis of OA. Type II collagen and aggrecan are the major components of the cartilage extracellular matrix, and their degradation underlies the structural failure of the tissue. Studies on transgenic mice have confirmed the central role of two groups of related metalloproteinases in cartilage matrix degradation: matrix metalloproteinases (MMPs), such as MMP-13, degrade type II collagen (Little et al., 2009), whereas adamalysin-like metalloproteinase with thrombospondin motifs (ADAMTS)-5 degrades aggrecan (Glasson et al., 2005; Stanton et al., 2005). These enzymes are thus considered to be potential therapeutic targets for OA, but their conserved catalytic domains have hampered development of sufficiently selective inhibitors to date.

We have adopted an alternative strategy of attempting to block cartilage degradation by increasing levels of the endogenous metalloproteinase inhibitor, tissue inhibitor of metalloproteinases (TIMP)-3, in the joint. The chondroprotective role of TIMP-3 is illustrated by studies showing that mice lacking the gene for *Timp3* develop accelerated OA as they age (Sahebjam et al., 2007) and, conversely, that recombinant TIMP-3 inhibits development of OA in a rat model of disease (Black et al., 2006). TIMP-3 levels are reduced in OA cartilage, although the mRNA levels are not altered (Morris et al., 2010).

We found that TIMP-3 levels are primarily controlled post-translationally and that TIMP-3 is readily endocytosed from the extracellular environment by the endocytic scavenger receptor low-density lipoprotein receptor–related protein 1 (LRP1) (Troeberg et al., 2008; Scilabra et al., 2013). We engineered mutants of TIMP-3 that do not bind to LRP1, and showed that they have a longer half-life in cartilage and protect cartilage better than wild-type TIMP-3 (Doherty et al., 2016). Sulfated glycosaminoglycans such as heparin, heparan sulfate, and pentosan polysulfate (PPS) are also able to inhibit cartilage degradation by inhibiting TIMP-3 binding to LRP1 and thus increasing extracellular levels of TIMP-3 (Troeberg et al., 2009, 2014; Scilabra et al., 2013). However, such sulfated glycosaminoglycans have poor pharmacokinetics and limited clinical scope. We thus sought to identify a small molecule inhibitor of TIMP-3 endocytosis that could serve as a lead compound for the development of novel OA therapeutics.

Yu et al. (2000) showed that TIMP-3 could be solubilized from extracellular matrices by suramin, a historic antiparasitic and antihelminthic drug. Here we show that suramin binds to TIMP-3 and inhibits its endocytosis by LRP1 and that suramin blocks degradation of both normal porcine cartilage and human OA cartilage in explant culture. We thus propose that suramin is a promising scaffold from which to develop a new type of therapeutic inhibitor to treat OA.

## Materials and Methods

### 

#### Materials.

C-terminally FLAG-tagged human TIMP-3 was expressed in human embryonic kidney 293 cells and purified as previously described (Troeberg et al., 2009). Receptor-associated protein was expressed in *Escherichia coli* and purified as described previously (Yamamoto et al., 2013). C-terminally FLAG-tagged ADAMTS-5 lacking the C-terminal thrombospondin domain was expressed in human embryonic kidney 293 cells and purified as previously described (Gendron et al., 2007). The catalytic domains of MMP-1 and MMP-3 were expressed in *E. coli* and purified as previously described (Suzuki et al., 1998; Chung et al., 2000).

The following suramin hexasodium salt and suramin analogs were from Tocris Bioscience (Bristol, UK): NF023 (8,8′-[carbonylbis(imino-3,1-phenylenecarbonylimino)]bis-1,3,5-naphthalene-trisulphonic acid, hexasodium salt), NF110 (4,4′,4′′,4′′′-[carbonylbis[imino-5,1,3-benzenetriylbis(carbonylimino)]]tetrakisbenzenesulfonic acid tetrasodium salt), NF157 [8,8′-[carbonylbis[imino-3,1-phenylenecarbonylimino(4-fluoro-3,1-phenylene)carbonylimino]]bis-1,3,5-naphthalenetrisulfonic acid hexasodium salt], NF279 (8,8′-[carbonylbis(imino-4,1-phenylenecarbonylimino-4,1-phenylenecarbonylimino)]bis-1,3,5-naphthalenetrisulfonic acid hexasodium salt), NF340 [4,4′-(carbonylbis(imino-3,1-(4-methyl-phenylene)carbonylimino))bis(naphthalene-2,6-disulfonic acid) tetrasodium salt], NF449 [4,4′,4′′,4′′′-[carbonylbis(imino-5,1,3-benzenetriyl-bis(carbonylimino))]tetrakis-1,3-benzenedisulfonic acid, octasodium salt], and NF546 [4,4′-(carbonylbis(imino-3,1-phenylene-carbonylimino-3,1-(4-methyl-phenylene)carbonylimino))-bis(1,3-xylene-*α*,*α*′-diphosphonic acid tetrasodium salt]. PPS was from Bene-PharmaChemie (Geretsried, Germany). Amphotericin B and M2 anti-FLAG antibody were from Sigma-Aldrich (Dorset, UK). TIMP-3 antibody (clone 183551, cat. no. MAB973) and mouse IgG1 isotype control (clone 11711, cat. no. MAB002) were from R&D Systems (Abingdon, UK), and anti-LRP1 (8G1, cat. no. ab20384) was from AbCam (Cambridge, UK). Quenched fluorescent substrates for MMPs and ADAMTS-5 were from Bachem (Bubendorf, Switzerland)

Dulbecco’s modified Eagle’s medium (DMEM), penicillin, streptomycin, amphotericin, HEPES, and trypsin-EDTA were from PAA Laboratories (Somerset, UK). Fetal calf serum (FCS) was from Gibco (Paisley, UK). Eppendorf Protein LoBind tubes were from VWR (East Grinstead, UK).

#### TIMP-3 Binding to Suramin and Analogs.

Glycosaminoglycan-binding ELISA plates (BD Life Sciences, Swindon, UK) were coated with suramin or its analogs (10 µg/ml in Tris-buffered saline, 18 hours, 25°C) (Mahoney et al., 2004) and wells were blocked with 0.2% gelatin in PBS (1 hour, 37°C). Wells were washed in PBS containing 0.1% Tween 20 after this and every subsequent step. Purified FLAG-tagged human TIMP-3 (0.4–50 nM) in blocking solution was applied to wells (3 hours, 37°C), and binding was detected with anti-FLAG M2 primary antibody and anti-mouse horseradish peroxidase–conjugated secondary antibody. 3,3′,5,5′-Tetramethylbenzidine (Becton Dickinson, Swindon, UK) substrate was added, the reaction was stopped when appropriate by adding 2 N H_2_SO_4_, and absorbance at 450 nm was measured using a FLUOstar Omega microplate reader (BMG Labtech, Aylesbury, Buckinghamshire, UK). Data (mean ± S.E., *n* = 3 technical repeats) were analyzed using Prism 7.0b software (GraphPad Software, La Jolla, CA) and EC_50_ values determined using a one-site specific binding model. Flat planar dimensions of suramin analogs were estimated using ICM-Pro software (Molsoft LLC, San Diego, CA).

#### TIMP-3 Binding to LRP1.

LRP1 (5 nM; BioMac, Leipzig, Germany) was coated (overnight, 4°C) onto medium-binding ELISA plates (Greiner Bio-One, Stonehouse, UK) in 20 mM HEPES, 150 mM NaCl, 5 mM CaCl_2_, and 0.05% Tween 20, pH 7.4. Wells were blocked with 10% bovine serum albumin (BSA) in Tris HCl, NaCl, and CaCl_2_ (TNC) buffer (50 mM Tris HCl, pH 7.5, 150 mM NaCl, 10 mM CaCl_2_, and 0.05% Brij 35). Wells were washed in TNC buffer containing 0.1% Tween 20 after this and every subsequent step. FLAG-tagged human TIMP-3 (0.4–50 nM), either alone or preincubated with suramin (200 μg/ml, 1 hour, 37°C), was applied to wells in TNC buffer containing 5% BSA (3 hours, 25°C). Binding was detected with anti-FLAG M2 primary antibody and anti-mouse horseradish peroxidase–conjugated secondary antibody in the same buffer. 3,3′,5,5′-Tetramethylbenzidine (Becton Dickinson) substrate was added, the reaction was stopped when appropriate by adding 2 N H_2_SO_4_, and absorbance at 450 nm was measured using a FLUOstar Omega microplate reader. Data (mean ± S.D., *n* = 3) were analyzed using Prism 7.0b software.

#### Cell and Cartilage Explant Culture.

HTB94 chondrosarcoma cells (American Culture Type Collection, Manassas, VA) were maintained in DMEM with 10% FCS, 100 U/ml penicillin, and 100 U/ml streptomycin at 37°C in 5% CO_2_.

Porcine and human cartilage explants and chondrocytes were maintained in DMEM with 10% FCS, 100 U/ml penicillin, 100 U/ml streptomycin, 2 mg/ml amphotericin B, and 10 mM HEPES at 37°C in 5% CO_2_. Porcine articular cartilage was dissected from metacarpophalangeal joints of 3- to 9-month-old pigs within 24 hours of euthanasia. Explants were prepared using a biopsy punch to ensure uniformity of size and rested for 48 hours before use. Chondrocytes were isolated by incubating dissected cartilage with type 2 collagenase (1 mg/ml; Worthington, Lakewood, NJ) in DMEM with 10% FCS (18 hours, 37°C). Cells were passed through a cell strainer and washed twice before plating.

Osteoarthritic human articular cartilage was obtained from patients undergoing knee replacement surgery. Tissue samples were obtained from the Oxford Musculoskeletal Biobank (Oxford, UK) and were collected with informed donor consent in full compliance with national and institutional ethical requirements, the United Kingdom Human Tissue Act, and the Declaration of Helsinki (HTA license 12217 and Oxford REC C 09/H0606/11). Human cartilage explants and chondrocytes were prepared as described for porcine cartilage above.

#### TIMP-3 Endocytosis Assays.

Cells (HTB94 chondrosarcoma cells, primary porcine chondrocytes, or human osteoarthritic chondrocytes) were plated overnight (6 × 10^5^ cells per well of a 12-well plate) in DMEM containing 10% FCS and washed three times in serum-free DMEM.

To evaluate endocytosis of exogenously added TIMP-3, cells were incubated with recombinant TIMP-3 (1 nM in 1.5 ml DMEM with 0.1% FCS) for 0–24 hours. Conditioned media were concentrated by precipitation with trichloroacetic acid [5% (v/v), 4°C, 18 hours]. After centrifugation (13,000 rpm, 4°C, 10 minutes), protein-containing pellets were resuspended in SDS sample buffer (30 µl), electrophoresed (7 µl) on a 10% polyacrylamide gel, and immunoblotted onto polyvinylidene fluoride. After blocking in 5% (m/v) BSA in Tris-buffered saline, TIMP-3 levels were analyzed using an M2 anti-FLAG antibody (Sigma-Aldrich), an alkaline phosphatase–conjugated anti-mouse secondary antibody (Promega, Southampton, UK), and a Western Blue stabilized substrate for alkaline phosphatase (Promega). Immunoblots were analyzed by densitometry using Phoretix 1D densitometry software (TotalLab, Newcastle-upon-Tyne, UK), and TIMP-3 remaining in the medium (mean ± S.D., *n* = 3) was calculated relative to pixel volume at a *t* of 0 hours (defined as 100%).

To evaluate accumulation of endogenous TIMP-3, cells were incubated with suramin (50–200 µg/ml) in serum-free DMEM for 30 hours. Media were harvested, precipitated with trichloroacetic acid, and analyzed by immunoblotting using a rabbit anti–TIMP-3 polyclonal antibody (AB6000; Millipore, Hertfordshire, UK) and an alkaline phosphatase–conjugated anti-rabbit secondary antibody (Promega). TIMP-3 in the medium (mean ± S.D., n = 3) was calculated relative to the pixel volume of untreated cells (defined as 1). 

#### mRNA Analysis.

HTB94 chondrosarcoma cells or primary human chondrocytes (1 × 10^6^ cells) were treated in triplicate with 0–250 µg/ml suramin in serum-free DMEM for 18–48 hours. Total RNA was isolated from cells using an RNeasy mini kit (Qiagen, Crawley, UK) and cDNA was synthesized (reverse transcriptase kit; Applied Biosystems, Foster City, CA). Levels of TIMP-1, TIMP-2, TIMP-3, LRP1, and RPLP0 internal reference mRNA were quantified by real-time polymerase (60S acidic ribosomal protein P0) chain reaction on a Corbett Rotor-Gene 6000 (Corbett Life Science, Mortlake, NSW, Australia) using TaqMan Fast Universal PCR Master Mix (Applied Biosystems) and TaqMan probes (Applied Biosystems) Hs01092512_g1 for TIMP-1, Hs00234278_m1 for TIMP-2, Hs00165949_m1 for TIMP-3, Hs00233856_m1 for LRP1, and Hs99999902_m1 for RPLP0. The ΔΔ threshold cycle values (mean ± S.D., *n* = 3 technical replicates) are shown relative to control untreated HTB94 cells (defined as 1).

#### Cartilage Explant Cultures.

The effects of suramin on cartilage degradation were assessed using porcine or human cartilage explants. Explants were rested for 48 hours after dissection and were washed with serum-free DMEM. Explants were then treated with retinoic acid (1 µM; Sigma-Aldrich), interleukin (IL)-1 (10 ng/ml; Peprotech, London, UK), and/or suramin (0–250 µg/ml) in serum-free DMEM for 48 hours.

Aggrecan degradation was analyzed by quantifying aggrecan fragments released into the conditioned media using the dimethylmethylene blue (DMMB) dye-binding assay (Farndale et al., 1986) (mean ± S.D., *n* = 3) and by immunoblotting with neo-epitope antibodies that recognize ADAMTS-cleaved but not intact aggrecan. For immunoblotting, conditioned media were deglycosylated by adding an equal volume of 200 mM sodium acetate and 50 mM Tris/HCl, pH 6.8, containing chondroitinase and *β*-endoglycosidase (0.05 U each; 18 hours, 37°C; Sigma-Aldrich). Aggrecan fragments were precipitated by adding five volumes of ice-cold acetone (18 hours, −20°C). After centrifugation (13,000 rpm, 10 minutes, 4°C), pellets were resuspended in SDS sample buffer, separated by electrophoresis on a 6% (v/v) polyacrylamide gel, and blotted onto polyvinylidene fluoride. Membranes were blocked with 5% (m/v) BSA in Tris-buffered saline and incubated with either a rabbit antibody that recognizes the AGEG neo-epitope generated by ADAMTS cleavage of the TAQE^1771^∼^1772^AGEG bond of aggrecan (Troeberg et al., 2008) or a mouse antibody that recognizes the ARGSV neo-epitope generated by ADAMTS cleavage of the NITEGE^373^∼^374^ARGSV bond (Hughes et al., 1995).

#### Cell Viability Assays.

HTB94 chondrosarcoma cells or human OA chondrocytes (10^3^ cells per well in 96-well plates) were plated overnight in DMEM with 10% FCS. Cells were washed in serum-free cartilage medium and treated for 48–72 hours with suramin (0–250 µg/ml) or sodium nitroprusside (10 mM) as a control to induce cell death. Cell viability was then assessed using the 3-(4,5-dimethylthiazol-2-yl)-5-(3-carboxymethoxyphenyl)-2-(4-sulfophenyl)-2*H*-tetrazolium CellTiter Cell Proliferation Assay (Promega) according to the manufacturer’s instructions (mean ± S.D., *n* = 3).

#### TIMP-3 Inhibition of Target Metalloproteinases.

The inhibition constant, *K*_i(app)_, for TIMP-3 inhibition of ADAMTS-5, MMP-1, and MMP-3 was determined under equilibrium kinetic conditions using the tight binding equation (Bieth, 1995). TIMP-3 (0.5 nM) was incubated with the target metalloproteinase (0.5 nM ADAMTS-5 or MMP-1) and/or suramin (0.05 µg/ml) or PPS (0.05 µg/ml) for 1 hour at 37°C, and residual enzyme activity against a quenched fluorescent substrate was determined. ADAMTS-5 activity was monitored using 20 µM *ortho*-aminobenzoyl-Thr-Glu-Ser-Glu∼Ser-Arg-Gly-Ala-Ile-Tyr-(*N*-3-[2,4-dinitrophenyl]-l-2,3-diaminopropionyl)-Lys-Lys-NH, and MMP-1 and MMP-3 activity was monitored using 1.5 µM 7-methoxycoumarin-4-yl) acetyl-Pro-Leu-Gly-Leu-(*N*-3-[2,4-dinitrophenyl]-l-2,3-diaminopropionyl)-Ala-Arg-NH_2_, as described previously (Troeberg et al., 2009). Steady-state velocities were determined using a Gemini microplate spectrofluorimeter (Molecular Devices, Wokingham, UK). *K*_i(app)_ (mean ± S.D., *n* = 3–5 independent experiments) was calculated using GraphPad Prism 7.0b to fit the data to the tight binding equation as follows (Bieth, 1995):
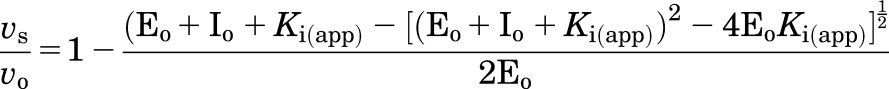
where *v*_o_ is the equilibrium rate of substrate hydrolysis in the absence of inhibitor, E_o_ is the total enzyme concentration, I_o_ is the total inhibitor concentration, and *K*_i(app)_ is the apparent inhibition constant.

## Results

### 

#### Suramin Inhibits the Endocytosis of TIMP-3 by the LRP1 Scavenger Receptor.

**Fig. 1.**
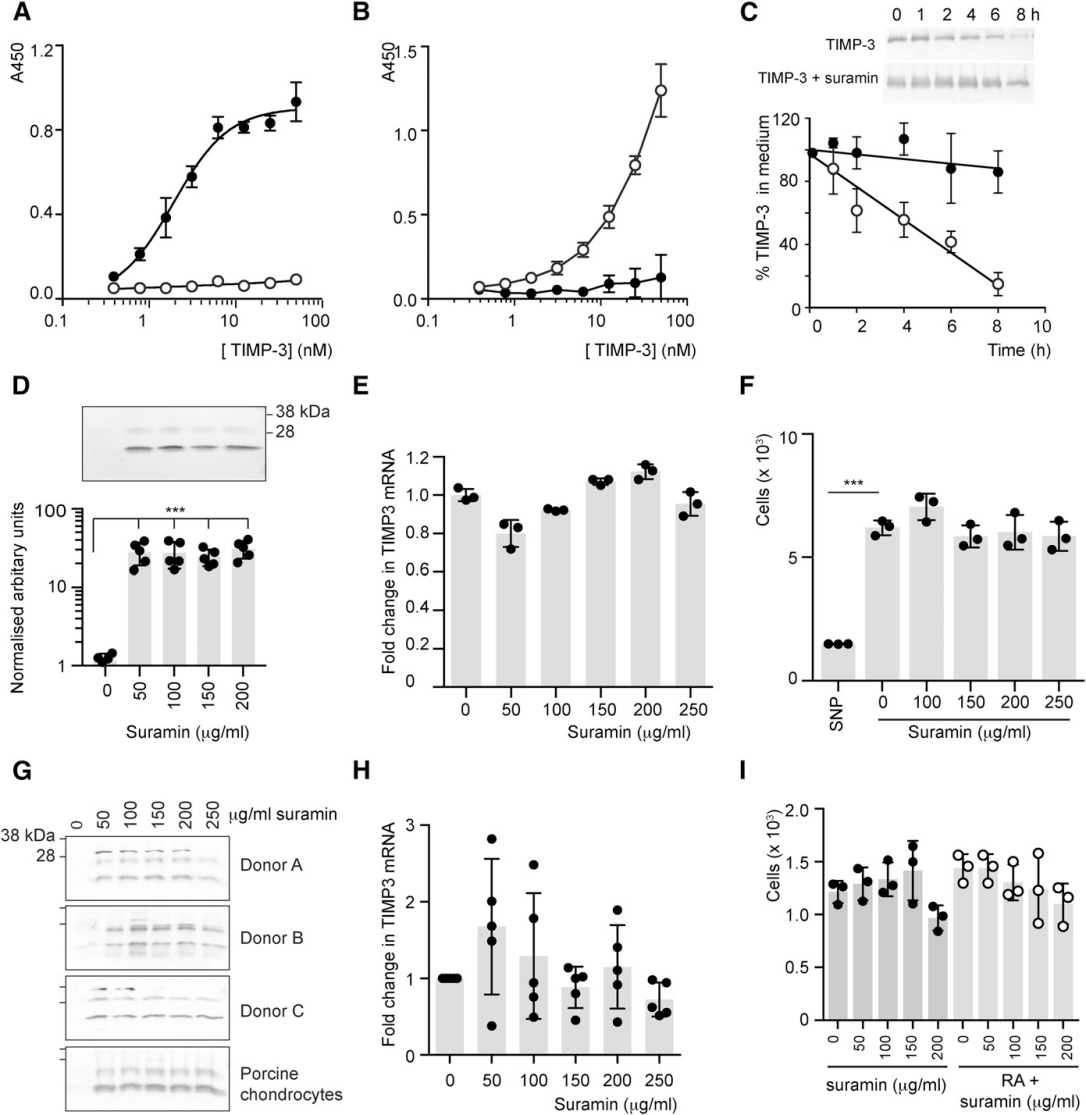
Suramin binds to TIMP-3 and inhibits its cellular endocytosis by LRP1. (A) Glycosaminglycan-binding 96-well plates were coated with suramin (10 µg/ml in PBS, filled circles) or PBS (open circles) and blocked in 0.2% gelatin in PBS. Wells were then incubated with TIMP-3 (0.4–50 nM) and binding was detected using an M2 anti-FLAG antibody (mean ± S.D., *n* = 3). (B) Medium-binding 96-well ELISA plates were coated with LRP1 (5 nM) and blocked with 10% BSA in TNC buffer. Wells were then incubated with TIMP-3 (0.4–50 nM), either alone (open circles) or preincubated with suramin (filled circles, 200 µg/ml, 1 hour, 37°C) and binding was detected using an M2 anti-FLAG antibody (mean ± S.D., *n* = 3). (C) HTB94 cells were incubated with recombinant TIMP-3 (1 nM) or TIMP-3 preincubated with suramin (200 µg/ml, 1 hour, 37°C) for 0–8 hours and TIMP-3 remaining in the medium was analyzed by immunoblotting and densitometry (mean ± S.D., *n* = 4). TIMP-3 (open circles) was taken up from the medium with a half-life of 4.0 ± 1.3 hours, whereas TIMP-3 preincubated with suramin (filled circles) was minimally endocytosed. (D) HTB94 chondrosarcoma cells were incubated with suramin (50–200 µg/ml) in serum-free DMEM for 30 hours. Conditioned media were concentrated by TCA precipitation and TIMP-3 levels were analyzed by immunoblotting and densitometry. Values are expressed relative to the amount of TIMP-3 in the medium of untreated cells, defined as 1 (mean ± S.D., *n* = 5; ***P ≤ 0.001 by one-way ANOVA with Bonferroni’s correction). (E) HTB94 chondrosarcoma cells were treated with suramin (0–250 µg/ml, 18 hours) and expression of TIMP-3 mRNA was analyzed by quantitative PCR relative to RPLP0. TIMP-3 expression in the absence of suramin was defined as 1 (mean ± S.D., *n* = 3, *P* > 0.05 by one-way ANOVA with Bonferroni’s correction). (F) HTB94 cells were treated with suramin (0–250 µg/ml) or sodium nitroprusside (10 mM) for 72 hours and cell viability was assessed using MTS (mean ± S.D., *n* = 3; ***P ≤ 0.001 by one-way ANOVA with Bonferroni’s correction). (G) Primary chondrocytes were isolated from human OA or porcine cartilage and incubated with suramin (0–250 µg/ml) in serum-free DMEM for 48 hours. Conditioned media were concentrated by TCA precipitation and TIMP-3 levels were analyzed by immunoblotting. (H) Human OA chondrocytes were treated with suramin (0–250 µg/ml, 48 hours) and expression of TIMP-3 mRNA was analyzed by quantitative PCR relative to RPLP0, with expression in the absence of suramin defined as 1 (*n* = 5 donors, mean ± S.D., *P* > 0.05 by one-way ANOVA with Bonferroni’s correction). (I) Human OA chondrocytes were treated with suramin (0–250 µg/ml) and/or retinoic acid (1 μM) for 48 hours and cell viability assessed using MTS (mean ± S.D., *n* = 3 donors, *P* > 0.05 by two-way ANOVA with Bonferroni’s correction). MTS, 3-(4,5-dimethylthiazol-2-yl)-5-(3-carboxymethoxyphenyl)-2-(4-sulfophenyl)-2*H*-tetrazolium; PCR, polymerase chain reaction; RA, retinoic acid; SNP, sodium nitroprusside; TCA, trichloroacetic acid.

As a first step to evaluating the effect of suramin on TIMP-3 endocytosis by the scavenger receptor LRP1, we examined TIMP-3 binding to immobilized suramin in a solid-phase binding assay. TIMP-3 bound strongly to suramin, with a *K*_D_ value of 1.9 ± 0.2 nM (Fig. 1A). TIMP-3 also bound strongly to immobilized LRP1 (Fig. 1B), and this binding was abolished when TIMP-3 was preincubated with suramin (200 µg/ml; Fig. 1B).

We previously showed that the rate of TIMP-3 endocytosis by LRP1 can be quantified by adding purified FLAG-tagged recombinant TIMP-3 (1 nM) to HTB94 chondrosarcoma cells and monitoring its disappearance from the medium (Doherty et al., 2016). In the absence of suramin, TIMP-3 was taken up from the medium with a half-life of 4.0 ± 1.3 hours (Fig. 1C). Preincubation of TIMP-3 with suramin (200 µg/ml, 1 hour, 37°C) markedly inhibited this uptake; more than 85% of TIMP-3 remained in the medium after 8 hours and a half-life could not be accurately calculated.

Suramin also inhibited endocytosis of endogenously expressed TIMP-3 in HTB94 cells. No TIMP-3 was detectable in the medium of untreated HTB94 cells, but TIMP-3 accumulated when cells were treated with suramin (50–200 µg/ml; Fig. 1D). This increase in TIMP-3 in the medium was not associated with any significant change in TIMP-3 mRNA levels (Fig. 1E). Suramin had no effect on cell viability, in contrast with sodium nitroprusside, a known cytotoxic agent (Fig. 1F).

Suramin similarly inhibited endocytosis of endogenously expressed TIMP-3 in primary chondrocytes isolated from human OA cartilage or normal porcine cartilage (Fig. 1G), without any significant change in TIMP-3 mRNA levels (Fig. 1H) or cell viability (Fig. 1I).

**Fig. 2.**
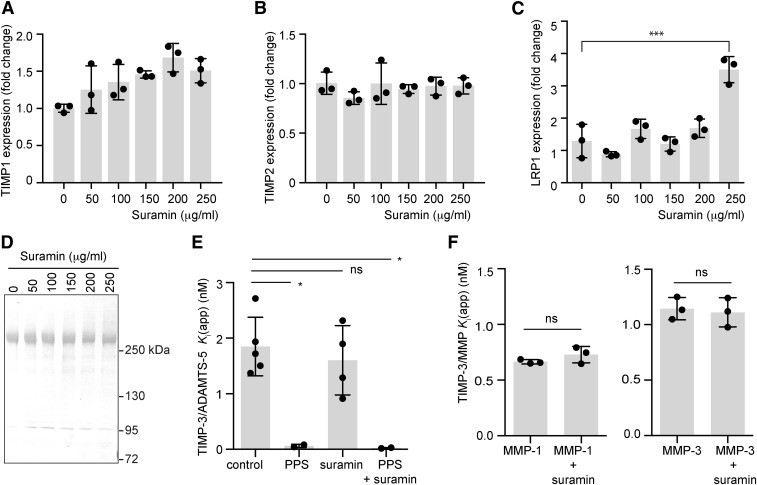
Suramin does not impair inhibitory activity of TIMP-3. (A) HTB94 cells were incubated with suramin (0–250 µg/ml, 18 hours) and expression of TIMP-1 mRNA was analyzed by quantitative PCR relative to RPLP0. TIMP-1 expression in the absence of suramin was defined as 1 (mean ± S.D., *n* = 3, *P* > 0.05 by one-way ANOVA with Bonferroni’s correction). (B) HTB94 cells were incubated with suramin (0–250 µg/ml, 18 hours) and expression of TIMP-2 mRNA was analyzed by quantitative PCR relative to RPLP0. TIMP-2 expression in the absence of suramin was defined as 1 (mean ± S.D., *n* = 3, *P* > 0.05 by one-way ANOVA with Bonferroni’s correction). (C) HTB94 cells were incubated with suramin (0–250 µg/ml, 18 hours) and expression of LRP1 mRNA was analyzed by quantitative PCR relative to RPLP0. LRP1 expression in the absence of suramin was defined as 1 (mean ± S.D., *n* = 3; ***P ≤ 0.001 by one-way ANOVA with Bonferroni’s correction). (D) HTB94 cells were treated with suramin (0–250 µg/ml, 30 hours), and conditioned media were concentrated by TCA precipitation and analyzed by immunoblotting using an 8G1 anti-LRP1 antibody. (E) ADAMTS-5 (0.5 nM) was incubated (1 hour, 37°C) with TIMP-3 (0.5–5 nM) and combinations of suramin (0.05 µg/ml) or PPS (0.05 µg/ml). Residual activity against a fluorescent peptide substrate was determined, and *K*_i(app)_ values (expressed in nM) were calculated from equilibrium rates of substrate hydrolysis using the tight binding equation (mean ± S.D., *n* = 4–5; *P ≤ 0.05 by one-way ANOVA with Bonferroni’s correction). (F) TIMP-3 (0.3–50 nM) was incubated (1 hour, 37°C) with MMP-1 (0.5 nM) or MMP-3 (1 nM) in the presence or absence of suramin (0.05 µg/ml). Residual activity against a fluorescent peptide substrate was determined, and *K*_i(app)_ values (expressed in nM) were calculated from equilibrium rates of substrate hydrolysis using the tight binding equation (mean ± S.D., *n* = 3; *P* > 0.05 by Student’s *t* test). ns, not significant.

Suramin had no effect on TIMP-1 or TIMP-2 mRNA levels in HTB94 cells (Fig. 2, A and B). Expression of LRP1 was unaffected by suramin at concentrations up to 200 µg/ml, although a 3-fold increase in expression was detected at 250 µg/ml (*P* < 0.001; Fig. 2C). Shedding of LRP1 ectodomain into the medium was also not affected by suramin (50–250 µg/ml; Fig. 2D).

#### Suramin Does Not Alter TIMP-3 Activity.

We evaluated whether TIMP-3 binding to suramin had any effect on its inhibition of target metalloproteinases in vitro. ADAMTS-5 is considered to be the primary aggrecan-degrading enzyme in murine (Glasson et al., 2005; Stanton et al., 2005) and human (Ismail et al., 2015) cartilage. TIMP-3 had an apparent affinity constant, *K*_i(app)_, of 2.03 ± 0.65 nM for ADAMTS-5 in the absence of suramin (Fig. 2E), in line with previous reports (Doherty et al., 2016). Preincubation of TIMP-3 with suramin had no significant effect on this affinity, with a *K*_i(app)_ of 1.60 ± 0.62 nM (Fig. 2E). In contrast, PPS reduced *K*_i(app)_ to a value too low to be calculated (Fig. 2E), as previously reported (Troeberg et al., 2012).

Suramin also had no effect on TIMP-3 affinity for MMP-1, with a *K*_i(app)_ of 0.66 ± 0.16 nM in the absence of suramin (Fig. 2F) and 0.65 ± 0.09 nM in the presence of suramin (Fig. 2F). Affinity for MMP-3 was similarly unaffected, with a *K*_i(app)_ of 1.14 ± 0.93 nM in the absence of suramin and 0.76 ± 0.09 nM in the presence of suramin.

#### Suramin Inhibits Cartilage Degradation.

**Fig. 3.**
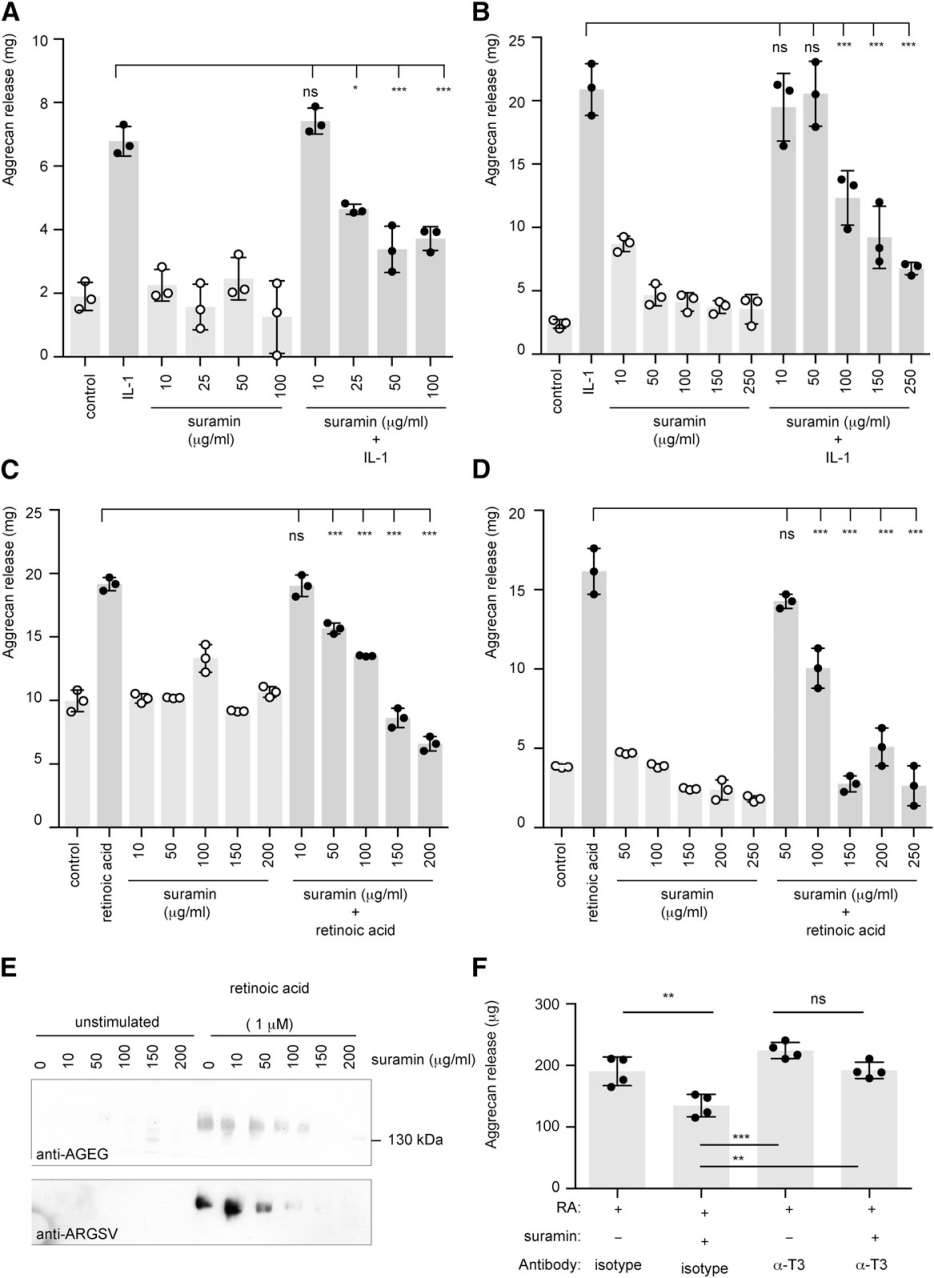
Suramin inhibits aggrecan degradation in retinoic acid–stimulated porcine and human cartilage explants. (A) Human OA cartilage explants were stimulated with IL-1 (10 ng/ml) in the presence of suramin (0–100 µg/ml) for 48 hours and degraded aggrecan fragments released into the conditioned medium quantified using the DMMB assay (mean ± S.D., *n* = 3; *P ≤ 0.05; ***P ≤ 0.001 by two-way ANOVA with Bonferroni’s correction). (B) Porcine cartilage explants were stimulated with IL-1 (10 ng/ml) in the presence of suramin (0–250 µg/ml) for 48 hours and degraded aggrecan fragments released into the conditioned medium were quantified using the DMMB assay (mean ± S.D., *n* = 3; ***P ≤ 0.001 by two-way ANOVA with Bonferroni’s correction). (C) Human OA cartilage explants were stimulated with retinoic acid (1 µM) in the presence of suramin (0–200 µg/ml) for 48 hours and degraded aggrecan fragments released into the conditioned medium were quantified using the DMMB assay (mean ± S.D., *n* = 3; ***P ≤ 0.001 by two-way ANOVA with Bonferroni’s correction). (D) Porcine cartilage explants were stimulated with retinoic acid (1 µM) in the presence of suramin (0–250 µg/ml) for 48 hours and degraded aggrecan fragments released into the conditioned medium were quantified using the DMMB assay (mean ± S.D., *n* = 3; ***P ≤ 0.001 by two-way ANOVA with Bonferroni’s correction). (E) Conditioned media from (D) were analyzed using neo-epitope antibodies that recognize the ^1772^AGEG or ^374^ARGSV termini generated by ADAMTS cleavage of aggrecan. (F) Porcine cartilage was incubated with retinoic acid (1 µM, 30 hours) in the presence of either a TIMP-3 antibody (MAB973, 50 µg/ml) or an isotype control (mouse IgG1, 50 µg/ml). Aggrecan degradation was quantified using the DMMB assay (mean ± S.D., *n* = 4; **P ≤ 0.01; ***P ≤ 0.001 by two-way ANOVA with Bonferroni’s correction). ns, not significant; RA, retinoic acid.

The effect of suramin on cartilage degradation was evaluated by measuring its effect on aggrecan release from cartilage explants in vitro. Explants of human knee cartilage were obtained at the time of joint replacement surgery for OA. Treatment of the explants with IL-1 or retinoic acid stimulated matrix catabolism, with the DMMB assay indicating a 2- to 3-fold increase in the amount of aggrecan released into the conditioned medium (Fig. 3, A and C). This aggrecan degradation was dose-dependently inhibited by suramin, with an IC_50_ of 62 ± 16 µg/ml (Fig. 3C). Similar efficacy was observed in cartilage from five other donors.

Suramin also effectively inhibited aggrecan release from IL-1 or retinoic acid–stimulated porcine cartilage explants, with an IC_50_ of 98 ± 9 µg/ml (Fig. 3D). Immunoblotting with the anti-ARGSV and anti-AGEG neo-epitope antibodies showed that suramin inhibited aggrecan degradation at the ADAMTS-susceptible NITEGE^373^∼^374^ARGSV and TAQE^1771^∼^1772^AGEG sites (Fig. 3E), indicating that aggrecanase activities were blocked.

To investigate whether protection by suramin was dependent on TIMP-3, we stimulated cartilage with retinoic acid and added suramin in combination with a TIMP-3 antibody or an isotype control antibody. In the presence of the isotope antibody, suramin significantly inhibited aggrecan release (Fig. 3F). However, in the presence of the anti–TIMP-3 antibody, suramin was unable to inhibit aggrecan degradation.

#### Suramin Analog NF279 Shows Improved Efficacy.

**Fig. 4.**
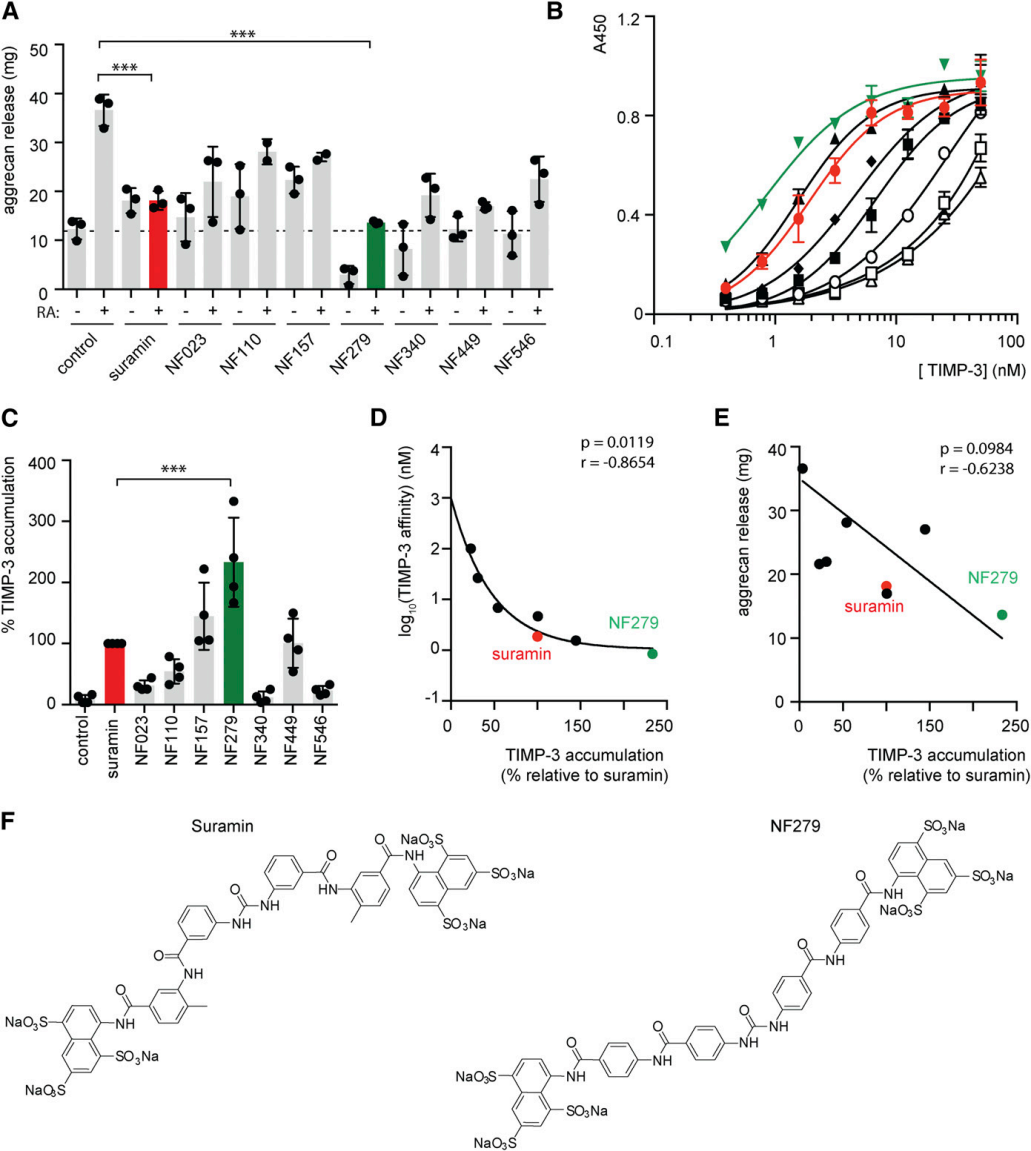
Suramin analog NF-279 shows improved ability to block TIMP-3 uptake and to protect cartilage. (A) Porcine cartilage explants were treated with retinoic acid (1 µM) and/or suramin analogs (200 µg/ml) for 48 hours. Cartilage degradation was assessed by quantifying aggrecan fragments (mean ± S.D., *n* = 3; ***P ≤ 0.001 by two-way ANOVA with Bonferroni’s correction) released into the medium using the DMMB assay. (B) Glycosaminglycan-binding 96-well plates were coated with 10 µg/ml suramin (red circles, EC_50_ = 1.90 ± 0.21 nM), NF279 (green triangles, EC_50_ = 0.85 ± 0.09 nM), NF110 (filled boxes, EC_50_ = 6.88 ± 0.96 nM), NF157 (filled triangles, EC_50_ = 1.55 ± 0.11 nM), NF449 (filled diamonds, EC_50_ = 4.66 ± 0.69 nM), NF023 (open circles, EC_50_ = 26.5 ± 7.5 nM), NF340 (open squares, EC_50_ > 100 nM), or NF546 (open triangles, EC_50_ > 100 nM) in PBS and blocked in 0.2% gelatin in PBS. Wells were then incubated with TIMP-3 (0.4–50 nM) and binding was detected using an M2 anti-FLAG antibody (mean ± S.E., *n* = 3 technical repeats). (C) HTB94 chondrosarcoma cells were cultured in the presence of suramin analogs (200 µg/ml) for 36 hours and TIMP-3 levels in the conditioned medium were evaluated by Western blotting and quantified by densitometry (mean ± S.D., *n* = 4, suramin defined as 100%; ***P ≤ 0.001 by one-way ANOVA with Bonferroni’s correction). (D) For each analog, TIMP-3 accumulation [from (C)] was plotted against the log_10_ of analog affinity for TIMP-3 [EC_50_ from (B)] and Pearson correlation coefficients were calculated using GraphPad Prism. (E) For each analog, TIMP-3 accumulation [from (C)] was plotted against aggrecan release [from (A)] and Pearson correlation coefficients were calculated using GraphPad Prism. (F) Structural formulae of suramin and its analog, NF279. RA, retinoic acid.

To further understand the mode of suramin action and to identify additional bioactive compounds, we tested the ability of seven commercially available suramin analogs (NF023, NF110, NF157, NF279, NF340, NF449, and NF546) to inhibit cartilage degradation. Porcine cartilage explants were stimulated with retinoic acid in the presence of 200 µg/ml suramin or a suramin analog for 48 hours, and cartilage degradation was quantified using the DMMB assay. NF279 showed markedly improved activity, inhibiting both retinoic acid–stimulated and unstimulated release of aggrecan fragments (Fig. 4A), with an IC_50_ of 15.6 ± 10 µg/ml.

The affinity of the analogs for TIMP-3 was evaluated using a solid-phase binding assay. NF279 had the highest affinity for TIMP-3, with a *K*_D_ value of 0.85 ± 0.09 nM (Fig. 1A). NF157 had similar affinity for TIMP-3 (*K*_D_ = 1.5 ± 0.11 nM) as was seen for suramin (*K*_D_ = 1.9 ± 0.2 nM, as in Fig. 1A), with the remaining analogs showing lower affinity than suramin.

The ability of the analogs to promote TIMP-3 accumulation was assessed by incubating HTB94 chondrosarcoma cells with suramin or the suramin analogs (200 µg/ml) for 36 hours and quantifying TIMP-3 levels in the medium by immunoblotting. The highest levels of TIMP-3 accumulated in cells treated with NF279 (233% ± 73% relative to suramin, defined as 100%; Fig. 4C). NF340 and NF546 showed minimal efficacy.

A strong exponential correlation was observed between the level of TIMP-3 accumulation (relative to suramin, defined as 100%) and the log_10_ of analog affinity for TIMP-3, with an *r* of −0.8654 (*P* = 0.0119; Fig. 4D). The level of TIMP-3 accumulation negatively correlated with aggrecan release, although this was nonsignificant (*r* = −0.6238, *P* = 0.0984; Fig. 4E).

## Discussion

OA has long been thought of a disease arising from passive wear and tear of the joints and as an inevitable consequence of aging. However, studies on knockout mice over the last decade have conclusively shown that OA is an active disease, in which various risk factors stimulate the cells in joint tissues to increase their catabolic activity. The roles of metalloproteinases in cartilage degradation are well established, with MMP-13 known to drive cleavage of type II collagen (Little et al., 2009) and ADAMTS-5 known to drive cleavage of aggrecan (Glasson et al., 2005; Stanton et al., 2005). The endogenous inhibitor of these enzymes, TIMP-3, is an important chondroprotective molecule, with *Timp3*-null animals developing accelerated OA (Sahebjam et al., 2007). The observation that TIMP-3 levels are reduced in human OA cartilage (Morris et al., 2010) led us to examine the molecular mechanisms regulating TIMP-3 levels, and it also led to the discovery that TIMP-3 is regulated post-translationally by endocytosis via the scavenger receptor LRP1 (Troeberg et al., 2008; Scilabra et al., 2013).

We recently engineered LRP1-resistant mutants of TIMP-3 and found that these have a longer half-life in cartilage and inhibit cartilage degradation at lower concentrations and for longer than wild-type TIMP-3 (Doherty et al., 2016). This illustrates that targeting the TIMP-3 endocytosis pathway is a potential strategy for inhibiting cartilage loss in OA. Administration of recombinant protein is unlikely to be a feasible therapeutic option for OA treatment, so we sought to develop small molecule inhibitors of TIMP-3 endocytosis that could increase levels of the endogenous inhibitor in cartilage. We show here that suramin, a polysulfonated naphthalene derivative of urea, binds to TIMP-3 and inhibits its endocytic uptake by cells through the LRP1 scavenger receptor, inhibiting degradation of aggrecan by ADAMTSs in cartilage.

TIMP-3 has a region of basic amino acids on its surface, and lysine and arginine residues in this area have been shown to mediate binding to sulfated proteoglycans (Lee et al., 2007; Troeberg et al., 2012) and LRP1 (Doherty et al., 2016). Suramin is likely to interact with this region of TIMP-3 via its negatively charged polysulfonated naphthylamine groups and hence to block interaction with LRP1. This basic region is on the opposite side of TIMP-3 to the one that interacts with target metalloproteinases, suggesting that suramin should not impair TIMP-3 inhibition of metalloproteinases. Indeed, we found that suramin had no effect on TIMP-3 affinity for ADAMTS-5, MMP-1, and MMP-3 in vitro. We previously found that sulfated molecules like heparin, heparan sulfate and PPS could increase TIMP-3 affinity for ADAMTS-4 and ADAMTS-5 (Troeberg et al., 2008, 2012, 2014), but this was not observed for suramin. This is likely because suramin is too short to simultaneously bind to basic regions on TIMP-3 and the enzymes and thus is unable to support formation of high-affinity trimolecular complexes.

Previous crystallography studies indicated that LRP1 ligands bind to the receptor through a pair of lysine residues 21 Å apart, which interact with two acidic pockets on sequential complementary repeats of LRP1 (Fisher et al., 2006). Mutation of several lysine residues on receptor-associated protein, a prototypic LRP1 ligand, reduce binding to LRP1, indicating that the extended charge landscape is important for orienting the two lysine residues that interact with the complementary repeat pockets (van den Biggelaar et al., 2011; Dolmer et al., 2013; Prasad et al., 2016). Our mutagenesis study of TIMP-3 supports this model, with LRP1 binding being reduced by mutation of several pairs of lysine residues separated by 21 ± 5 Å (Doherty et al., 2016). In an extended flat planar conformation, suramin has an estimated maximal length of 38 Å. Although the molecule is likely to adopt numerous conformations in solution, this indicates it is of sufficient length for the two clusters of sulfonate groups on either end of the suramin molecule to bind to the LRP1-interacting dilysine motif on TIMP-3 and thus to block TIMP-3 interaction with the endocytic receptor.

We evaluated seven commercially available suramin analogs and found that NF279 exhibited an improved chondroprotective activity, with a 6-fold improved IC_50_ value of 15 µg/ml (11.6 μM). This correlated with an increased affinity for TIMP-3 and accumulation of higher TIMP-3 levels in NF279-treated cells. The structure of NF279 is very similar to that of suramin, with two clusters of sulfonate groups at each end of the molecule (Fig. 4F) and an estimated maximal length of 39 Å in an extended planar conformation, suggesting that it has a similar binding mode to suramin. Shorter analogs (e.g., NF340 with an estimated maximal length of 23 Å) and or analogs with fewer sulfonate groups (e.g., NF546) were less effective at protecting cartilage and supporting TIMP-3 accumulation. NF279 may adopt a more extended conformation in solution than suramin, since the phenyl links in the middle of the compound are in *para*, rather than *meta*, orientations. This may enable NF279 to more effectively bridge LRP1-interacting residues on TIMP-3. Further analogs will need to be evaluated to improve understanding of the structure-activity relationship of the suramin scaffold.

Suramin has been shown to ameliorate cartilage damage in a rat inflammatory arthritis model of rheumatoid arthritis (Sahu et al., 2012), with reduced levels of proinflammatory cytokines in the plasma and joints of treated animals. Although OA is associated with metalloproteinase degradation of cartilage extracellular matrix components, rheumatoid arthritis is an inflammatory disease driven by proinflammatory cytokines, such as tumor necrosis factor (TNF) and IL-1. TIMP-3 inhibits release of active TNF by inhibiting the activity of the metalloproteinase A Disintegrin and Metalloproteinase 17 (ADAM17, or TNF*α*-converting enzyme) (Mohammed et al., 2004), so we hypothesize that suramin also protected cartilage in this inflammatory arthritis model by blocking endocytosis of TIMP-3 by LRP1. Although the pathologic role of inflammation in OA is speculative rather than proven, suramin’s ability to inhibit inflammation as well as metalloprotease-driven cartilage breakdown is likely to further augment its chondroprotective effect.

Suramin has been shown to have several biologic effects, so it is possible that suramin protects cartilage through molecular mechanisms other than inhibiting TIMP-3 endocytosis. Since its development in 1916, suramin has been used to treat human infection with protozoan *Trypanosoma* and helminthic *Onchocerca* parasites (Hawking, 1978; Voogd et al., 1993). Its mechanism of antiparasitic action is unclear. Suramin inhibits several trypanosomal glycolytic enzymes in vitro (Misset and Opperdoes, 1987) and reduces ATP generation in vivo (Fairlamb and Bowman, 1980). Suramin has been shown to inhibit MMP-9 activity (Taniguti et al., 2012), and we found that it can also inhibit MMP-2 activity in vitro (data not shown). This is unlikely to contribute to the chondroprotection we observed in this study, in which cartilage degradation in the first 1–3 days after explant stimulation was mediated by ADAMTSs, with MMP-dependent degradation only evident after 14–21 days (Pratta et al., 2003; Lim et al., 2010). Suramin is also known to antagonize ATP purinergic signaling through the P_2_X receptor (Dunn and Blakeley, 1988). NF279 is less effective than suramin at inhibiting purinergic signaling in chondrocytes (Varani et al., 2008), so its increased chondroprotection argues against P_2_X antagonism being the primary mechanism of suramin chondroprotective action. It is likely that suramin will have additional mechanisms of action in vivo, but our observation that the protective effects of suramin were inhibited by a TIMP-3 blocking antibody suggest that TIMP-3 is required for suramin’s chondroprotective effect and that analogs with improved ability to block TIMP-3 uptake will have improved ability to protect cartilage.

Suramin is not orally bioavailable (Voogd et al., 1993) and violates several of Lipinski’s rule of 5. It has a molecular mass of 1297 g/ml, more than five hydrogen bond donors, and more than 10 hydrogen bond donors. Patients with OA are commonly treated with intra-articular injection of corticosteroids, indicating that intra-articular administration of suramin is feasible. Further development of orally bioavailable suramin analogs could be pursued by “lead-hopping” to a smaller chemical series once structure-activity relationships are more fully understood.

Suramin’s clinical use has been limited by its adrenal toxicity. Although suramin did not affect chondrocyte viability at the concentrations and durations tested here, its toxicity profile would prevent its systemic use in patients with OA. It would also be undesirable to increase TIMP-3 levels systemically, since metalloproteinase activity is required for numerous physiologic processes, including wound healing and angiogenesis. It would thus be necessary to target suramin derivatives to cartilage, for example, through use of a cartilage-targeting peptide (Rothenfluh et al., 2008). Our limited scan of suramin analogs identified a more effective analog and suggested some basic structure-activity requirements for activity, indicating the potential for engineering more effective variants with improved safety profiles. We thus propose suramin as a promising scaffold for the development of novel therapeutics to target osteoarthritic cartilage loss.

## Abbreviations

ADAMTS: adamalysin-like metalloproteinase with thrombospondin motifs
BSA: bovine serum albumin
DMEM: Dulbecco’s modified Eagle’s medium
DMMB: dimethylmethylene blue
FCS: fetal calf serum
IL: interleukin
LRP1: low-density lipoprotein receptor–related protein 1
MMP: matrix metalloproteinase
NF023: 8,8′-[carbonylbis(imino-3,1-phenylenecarbonylimino)]bis-1,3,5-naphthalene-trisulphonic acid, hexasodium salt)
NF110: 4,4′,4′′,4′′′-[carbonylbis[imino-5,1,3-benzenetriylbis(carbonylimino)]]tetrakisbenzenesulfonic acid tetrasodium salt
NF157: 8,8′-[carbonylbis[imino-3,1-phenylenecarbonylimino(4-fluoro-3,1-phenylene)carbonylimino]]bis-1,3,5-naphthalenetrisulfonic acid hexasodium salt
NF279: 8,8′-[carbonylbis(imino-4,1-phenylenecarbonylimino-4,1-phenylenecarbonylimino)]bis-1,3,5-naphthalenetrisulfonic acid hexasodium salt
NF340: 4,4′-(carbonylbis(imino-3,1-(4-methyl-phenylene)carbonylimino))bis(naphthalene-2,6-disulfonic acid) tetrasodium salt
NF449: 4,4′,4′′,4′′′-[carbonylbis(imino-5,1,3-benzenetriyl-bis(carbonylimino))]tetrakis-1,3-benzenedisulfonic acid, octasodium salt
NF546: 4,4′-(carbonylbis(imino-3,1-phenylene-carbonylimino-3,1-(4-methyl-phenylene)carbonylimino))-bis(1,3-xylene-*α*,*α*′-diphosphonic acid tetrasodium salt
OA: osteoarthritis
PPS: pentosan polysulfate
RPLP0: 60S acidic ribosomal protein P0
TIMP: tissue inhibitor of metalloproteinases
TNC: Tris HCl, NaCl, and CaCl_2_
TNF: tumor necrosis factor
